# Sex Differences in Use of Low Tidal Volume Ventilation in COVID-19—Insights From the PRoVENT–COVID Study

**DOI:** 10.3389/fmed.2021.780005

**Published:** 2022-01-03

**Authors:** Pien Swart, Sunny G. L. H. Nijbroek, Frederique Paulus, Ary Serpa Neto, Marcus J. Schultz

**Affiliations:** ^1^Department of Intensive Care, Amsterdam University Medical Center, Location ‘Academic Medical Center’, Amsterdam, Netherlands; ^2^Department of Anaesthesiology, Amsterdam University Medical Center, Location ‘Academic Medical Center’, Amsterdam, Netherlands; ^3^Australian and New Zealand Intensive Care Research Centre, Monash University, Melbourne, VIC, Australia; ^4^Department of Critical Care Medicine, Hospital Israelita Albert Einstein, São Paulo, Brazil; ^5^Mahidol Oxford Tropical Medicine Research Unit (MORU), Mahidol University, Bangkok, Thailand; ^6^Nuffield Department of Medicine, University of Oxford, Oxford, United Kingdom

**Keywords:** lung protective ventilation, low tidal volume ventilation (LTVV), sex, gender, COVID-19, intensive care unit, critical care, mechanical ventilation

## Abstract

The purpose of this study was to compare and understand differences in the use of low tidal volume ventilation (LTVV) between females and males with acute respiratory distress syndrome (ARDS) related to coronavirus disease 2019 (COVID-19). This is a *post-hoc* analysis of an observational study in invasively ventilated patients with ARDS related to COVID-19 in 22 ICUs in the Netherlands. The primary endpoint was the use of LTVV, defined as having received a median tidal volume (V_T_) ≤6 ml/kg predicted body weight (PBW) during controlled ventilation. A mediation analysis was used to investigate the impact of anthropometric factors, next to the impact of sex *per se*. The analysis included 934 patients, 251 females and 683 males. All the patients had ARDS, and there were no differences in ARDS severity between the sexes. On the first day of ventilation, females received ventilation with a higher median V_T_ compared with males [6.8 (interquartile range (IQR) 6.0–7.6 vs. 6.3 (IQR 5.8–6.9) ml/kg PBW; *p* < 0.001]. Consequently, females received LTVV less often than males (23 vs. 34%; *p* = 0.003). The difference in the use of LTVV became smaller but persisted over the next days (27 vs. 36%; *p* = 0.046 at day 2 and 28 vs. 38%; *p* = 0.030 at day 3). The difference in the use LTVV was significantly mediated by sex *per se* [average direct effect of the female sex, 7.5% (95% CI, 1.7–13.3%); *p* = 0.011] and by differences in the body height [average causal mediation effect, −17.5% (−21.5 to −13.5%); *p* < 0.001], but not by the differences in actual body weight [average causal mediation effect, 0.2% (−0.8 to 1.2%); *p* = 0.715]. In conclusion, in this cohort of patients with ARDS related to COVID-19, females received LTVV less often than males in the first days of invasive ventilation. The difference in the use of LTVV was mainly driven by an anthropometric factor, namely, body height. Use of LTVV may improve by paying attention to correct titration of V_T_, which should be based on PBW, which is a function of body height.

## Introduction

Coronavirus disease 2019 (COVID-19) pandemic continues to have a relentless impact on the healthcare systems worldwide. Critical care systems are overloaded as many patients with COVID-19 develop acute respiratory failure requiring admission to a hospital for supplementary oxygen. A substantial proportion of these patients need admission to an intensive care unit (ICU) for ventilatory support (1, 2). Lung–protective ventilation, including the use of a low tidal volume (V_T_), is recommended in patients with acute respiratory distress syndrome (ARDS) (3, 4) and there is growing evidence that the use of low-V_T_ ventilation (LTVV) also benefits patients with ARDS related to COVID-19 (5, 6).

Differences between females and males with regard to the use of LTVV have been described in surgery patients during general anesthesia (7–12) as well as critically ill patients in the ICU—and irrespective of the presence of ARDS (13–16, 44). It is uncertain if the sex difference in the use of LTVV also exists in patients with COVID-19. Use of LTVV might be limited in these patients because, due to the large numbers of patients requiring respiratory support, ventilation may need to be provided by healthcare professionals with much less experience in invasive ventilation, and thus also in the use of LTVV—it is uncertain whether this translates into sex differences.

To compare ventilation management with respect to LTVV in females vs. males, we reassessed the database of a conveniently-sized national multicenter study named “PRactice of VENTilation in patients with COVID-19” (PRoVENT–COVID) (5), a study that focused on ventilator settings and ventilation parameters in the first 4 calendar days of ventilation. Next to the hypothesis that the use of LTVV differs between the sexes, we also tested the hypothesis that differences in LTVV use are driven by anthropometric differences, i.e., differences in height and weight between the sexes, more than by sex *per se*.

## Materials and Methods

### Design, Setting, and Participants

Secondary analysis of the database from the PRoVENT–COVID study, an investigator-initiated, national, multicenter, observational study in 22 ICUs in the Netherlands in the first 3 months of the national outbreak (5).

The protocol of the study of PRoVENT–COVID was approved by the institutional review boards of each participating hospital—need for individual patient informed consent was waived seen the observational design of the investigation. The PRoVENT–COVID study was registered at clinicaltrial.gov under the identifier NCT04346342.

Consecutive patients aged 18 years or older were enrolled if admitted to an ICU in one of the participating hospitals and having had received invasive ventilation for acute respiratory failure due to COVID-19, which had to be confirmed by RT–PCR. The PRoVENT–COVID study excluded SARS–CoV−2 infected patients that received ventilation for other reasons than COVID-19, e.g., patients that received ventilation for post-operative ventilation.

### Data Collection and Analysis

Demographics, home medication, comorbidities, and disease severity scores were collected at baseline. The Berlin definition for ARDS was used to determine whether a patient had ARDS, and for ARDS severity (17).

Detailed information regarding ventilation management was captured in the first 4 calendar days of invasive ventilation at fixed time points every 8 h. Pulmonary and extrapulmonary events were captured up to hospital discharge, with a maximum of 28 days. Outcomes, such as intubation and life status, were collected till day 90.

We used the following equations:


(1) $$\begin{array}{l} \begin{array}{c} {\text{V}}_{\text{T}}{\text{ normalized to predicted body weight (PBW) (V}}_{\text{T,PBW}}\text{)} \end{array}\\ {\text{ [ml/kg] = absolute V}}_{\text{T}}\text{ (ml)/PBW (kg) } \end{array}$$

(18);


(2a) $$\begin{array}{rcl} \text{PBW in females }\left(\text{kg}\right) & = & 45.5+0.91^{*}\left(\text{height }\left[\text{cm}\right]-152.4\right); \end{array}$$

(2b) $$\begin{array}{rcl} \text{and PBW in males }\left(\text{kg}\right) & = & 50.0+0.91^{*}\left(\text{height }\left[\text{cm}\right]-152.4\right); \end{array}$$

(3) $$\begin{array}{l} \begin{array}{c} {\text{V}}_{\text{T}}{\text{ normalized to actual body weight (ABW) (V}}_{\text{T,ABW}}\text{)} \end{array}\\ \begin{array}{c} {\text{[ml/kg] = absolute V}}_{\text{T}}\text{ (ml)/ABW (kg)} \end{array}\; \end{array}$$

(4) $$\begin{array}{l} {\text{driving pressure (ΔP) [cm H}}_{2}\text{O] = peak pressure (Ppeak)}\\ {\text{[cm H}}_{2}{\text{O] − PEEP [cm H}}_{2}\text{O]; and}\\ {\text{respiratory system compliance (Crs) [ml/cm H}}_{2}\text{O]}\\ \begin{array}{c} {\text{= Absolute V}}_{\text{T}}{\text{ (ml)/ΔP [cm H}}_{\text{2}}\text{O]} \end{array} \end{array}$$

### Study Endpoints

The primary endpoint was the use of LTVV in the first 4 calendar days of invasive ventilation. Secondary endpoints were other key ventilation parameters, including absolute V_T_, V_T,ABW_ and V_T,PBW_, PEEP, ΔP, and Crs.

### Power Calculation

The PRoVENT–COVID study contains a conveniently sized cohort of patients. We did not perform a formal power calculation; the sample size was based on the number of patients available in the database. With 1,000 patients, the study has >80% power to detect an absolute difference ranging from 9 and 15% in the use of LTVV considering a use rate of 50% in the female patients as shown previously (16).

### Statistical Analysis

No assumptions were made for missing data. As the first calendar day was a flexible day that lasted from the moment of intubation and start of ventilation in the participating ICU and in theory could last from 1 min to 23 h and 59 min, we merged the first and second calendar day, which was then named “day 1.” The following calendar days were named “day 2” and “day 3.” As the ventilation strategy and settings may vary substantially in the first hour of intubation, we also ignored the first available V_T_, i.e., collected within 1 h of intubation.

Data are reported as numbers and proportions for categorical variables, and as medians with interquartile ranges (IQRs) for continuous variables. In addition, we also provided the 90% range for V_T_. For baseline characteristics, the sexes were compared using the Fisher exact test for categorical variables, and the Wilcoxon rank-sum test for continuous variables. In all the analyses, males are used as the reference.

Ventilation parameters per day are presented in cumulative distribution plots, and in line graphs with error bars. In the distribution plots, vertical dotted lines represent the ideal cutoff for each parameter, and horizontal dotted lines the respective proportion of patients reaching each cutoff. All the ventilatory variables were aggregated per day and reported as such. For this, we calculated the mean of each ventilatory parameter per patient per day. In the tables, continuous variables were reported as medians of the means per each patient.

Patients were classified as having received LTVV, if the mean V_T,PBW_ was ≤6 ml/kg during the controlled ventilation. For day 1, we ignored the breath in the first hour of ventilation, as this breath could have not been adjusted to achieve LTVV, e.g., in patients who started ventilation in the emergency department. Breaths collected under pressure support ventilation were also ignored, as were a breath that was collected at the moment spontaneous breathing activity was likely. This was the case if the measured (total) RR exceeded the set RR >2 breaths per min.

To further assess if sex is associated with differences in V_T_ an unadjusted mixed-effects linear regression model was used to extract the risk difference among the sexes. All analyses were performed using multilevel (patients nested in hospitals), mixed modeling with hospitals as a random effect to account for within-center clustering. Two *P*-values were reported in the graphs: (1) *P*-value for sex differences, reflecting the overall test for difference between sex across the days; and (2) *p-*values for the sex × day interaction, evaluating if change over time differed between the sexes.

The proportions of patients having had received LTVV are described and visualized in pie charts. An unadjusted mixed-effect generalized linear model was used to extract the risk difference for LTVV use.

To investigate whether differences in the use of LTVV between females and males are mediated by body height and ABW, a mixed-effect mediation model was used. In the mediation analysis, we assessed the individual impact of body height and ABW as potential mediators for the difference in the use of LTVV between sex. Mediators are variables that are affected by group assignment and that subsequently can affect the outcome. Therefore, mediators are on the causal pathway of the relation between group and outcome, at least partly explaining the effects of the group on the outcome. For the mediation models, the following estimates are described: (1) the total effect (estimates the total effect of sex on ventilation); (2) the average causal mediation effect [ACME, explains how much of the effect of sex on ventilation is explained by the mediator (height or weight)]; and (3) the average direct effect (ADE, explains how much of the effect of sex on ventilation is still explained by sex after considering the effect of the mediator). For this model, Quasi–Bayesian 95% CI were estimated after 10,000 simulations. The mediation models included day and centers as a random effects.

All the analyses were done in R version 4.0.2 and the significance level was set at 0.05.

## Results

### Patients

**Figure 1:**
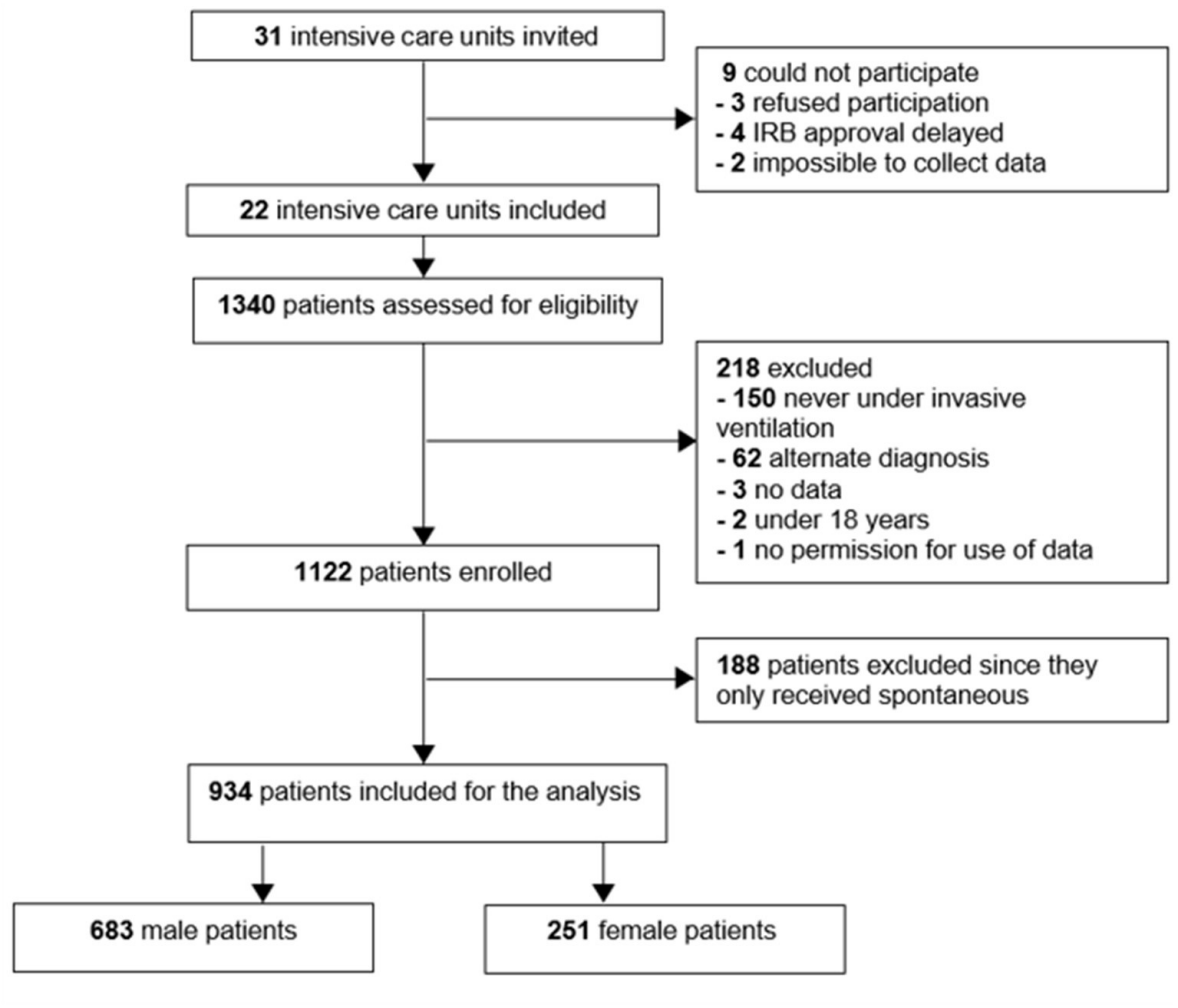
Study flowchart.

Of 1,122 eligible patients, 188 patients did not receive controlled ventilation at any time point data were collected for this study, leaving us with 934 fully analyzable patients, 251 females and 683 males (Figure 1). Males had a higher median body height and also a higher median ABW. Aside from differences in baseline APACHE II scores, plasma creatinine, and use of statins and inhalation corticosteroids at home, there were no differences at baseline between the sexes (Table 1). Other severity scores and ARDS severity were comparable between females and males (Table 1). Compared with females, males had a higher mortality rate and a longer duration of ventilation (Table 2).

**Table 1:** Baseline characteristics of patient.

	**Overall**	**Females**	**Males**	***p***
Number of patients	934	251	683	
Age, years	65.0 [57.0, 72.0]	64.0 [55.0, 71.5]	65.0 [57.0, 72.0]	0.177
Weight, kg	86.0 [77.3, 96.4]	80.0 [70.0, 90.0]	89.0 [80.0, 98.2]	<0.001
Height, cm	176.0 [170.0, 183.0]	165.0 [162.0, 170.0]	180.0 [174.0, 185.0]	<0.001
BMI, kg/m^2^	27.8 [25.2, 30.9]	28.4 [25.9, 32.3]	27.6 [25.2, 30.1]	0.002
Intubation at admission	152 (16.3)	38 (15.1)	114 (16.7)	0.618
NIV before intubation	77 (9.2)	23 (10.0)	54 (8.9)	0.688
Duration of NIV	7.5 [2.0, 18.1]	5.0 [1.8, 11.5]	8.0 [2.2, 24.0]	0.327
CT before intubation	326 (36.2)	97 (40.8)	229 (34.6)	0.099
% affected lung parenchyma on CT				0.682
0%	14 (4.3)	4 (4.1)	10 (4.3)	
25%	103 (31.4)	27 (27.8)	76 (32.9)	
50%	99 (30.2)	35 (36.1)	64 (27.7)	
75%	93 (28.4)	26 (26.8)	67 (29.0)	
100%	19 (5.8)	5 (5.2)	14 (6.1)	
X-ray before intubation	485 (85.7)	122 (85.3)	363 (85.8)	0.891
Number of affected quadrants				0.335
1	38 (7.8)	7 (5.9)	31 (8.5)	
2	114 (23.5)	34 (28.6)	80 (21.9)	
3	135 (27.8)	35 (29.4)	100 (27.3)	
4	198 (40.8)	43 (36.1)	155 (42.3)	
Pneumothorax	4 (1.8)	2 (3.8)	2 (1.2)	0.238
SAPS II	36.0 [29.0, 43.5]	35.0 [31.0, 43.5]	36.0 [29.0, 43.2]	0.573
APACHE II	16.0 [14.0, 21.0]	15.0 [12.0, 20.0]	17.0 [14.0, 22.0]	0.039
APACHE IV	56.0 [45.0, 70.0]	57.0 [46.0, 69.2]	56.0 [44.0, 70.0]	0.460
SOFA	7.0 [6.0, 10.0]	7.0 [6.0, 9.2]	7.0 [6.0, 10.0]	0.160
ARDS severity				0.386
Mild	188 (20.4)	51 (20.7)	137 (20.3)	
Moderate	630 (68.4)	162 (65.9)	468 (69.3)	
Severe	103 (11.2)	33 (13.4)	70 (10.4)	
**Co-existing disorders**
Arterial hypertension	310 (33.2)	72 (28.7)	238 (34.8)	0.085
Heart failure	37 (4.0)	7 (2.8)	30 (4.4)	0.345
Diabetes	214 (22.9)	56 (22.3)	158 (23.1)	0.861
Chronic kidney disease	39 (4.2)	10 (4.0)	29 (4.2)	1.000
Baseline creatinine, μmol/L^*^	77.0 [62.0, 98.5]	63.5 [51.8, 78.0]	82.0 [68.0, 105.0]	<0.001
Liver cirrhosis	3 (0.3)	2 (0.8)	1 (0.1)	0.178
COPD	72 (7.7)	20 (8.0)	52 (7.6)	0.890
Active hematological malignancy	13 (1.4)	3 (1.2)	10 (1.5)	1.000
Active solid tumor malignancy	26 (2.8)	10 (4.0)	16 (2.3)	0.182
Neuromuscular disease	4 (0.4)	2 (0.8)	2 (0.3)	0.294
Immunosuppression	20 (2.1)	5 (2.0)	15 (2.2)	1.000
**Home medication**
Systemic corticosteroids	34 (3.6)	8 (3.2)	26 (3.8)	0.844
Inhalation corticosteroids	105 (11.2)	40 (15.9)	65 (9.5)	0.007
ACE inhibitor	155 (16.6)	37 (14.7)	118 (17.3)	0.374
ARB II	106 (11.3)	22 (8.8)	84 (12.3)	0.162
Beta blocker	171 (18.3)	37 (14.7)	134 (19.6)	0.104
Insulin	68 (7.3)	20 (8.0)	48 (7.0)	0.670
Metformin	148 (15.8)	33 (13.1)	115 (16.8)	0.189
Statin	284 (30.4)	62 (24.7)	222 (32.5)	0.025
Calcium channel blocker	165 (17.7)	41 (16.3)	124 (18.2)	0.562

*Data are median (quartile 25%–quartile 75%) or no (%). Percentages may not total 100 because of rounding*.

* *Most recent measurement in 24 h before intubation, or at ICU admission under invasive ventilation*.

*APACHE, Acute Physiology and Chronic Health Evaluation; SAPS, Simplified Acute Physiology Score; SOFA, Sequential Organ Failure Assessment; ARDS, acute respiratory distress syndrome*.

**Table 2:** Outcome.

	**Overall**	**Females**	**Males**	***p***
Number of patients	934	251	683	
Ventilatory free days at 28 days	2.0 [0.0, 16.0]	9.0 [0.0, 18.0]	0.0 [0.0, 15.0]	0.001
Extubation	545 (58.8)	159 (63.3)	386 (57.1)	0.098
Duration of ventilation, days	14.0 [8.0, 23.0]	12.0 [7.0, 20.8]	15.0 [8.0, 24.0]	0.012
Duration of ventilation in survivors at day 28	16.0 [10.0, 28.0]	13.0 [9.0, 23.0]	17.0 [10.0, 29.0]	0.003
Tracheostomy	154 (16.6)	32 (12.8)	122 (18.1)	0.059
Reintubation	118 (12.8)	38 (15.3)	80 (11.9)	0.184
Pneumothorax	8 (0.9)	1 (0.4)	7 (1.1)	0.690
Thromboembolic complications^*^	266 (28.5)	65 (25.9)	201 (29.4)	0.326
Acute kidney injury^**^	421 (45.2)	100 (40.0)	321 (47.1)	0.054
Renal replacement therapy	173 (18.5)	35 (13.9)	138 (20.2)	0.029
ICU length of stay, days	15.0 [9.0, 27.0]	14.0 [9.0, 24.0]	16.0 [9.0, 27.5]	0.094
In survivors, days	18.0 [11.0, 30.0]	16.0 [10.0, 27.0]	18.0 [11.0, 31.0]	0.067
Hospital length of stay, days	24.0 [14.0, 37.0]	22.0 [14.0, 36.0]	24.0 [14.0, 39.0]	0.408
In survivors, days	29.5 [20.0, 44.0]	27.0 [20.0, 39.0]	30.0 [20.0, 46.0]	0.062
ICU mortality	301 (33.0)	66 (27.2)	235 (35.2)	0.026
Hospital mortality	310 (36.1)	67 (29.9)	243 (38.3)	0.029
d7 mortality	97 (10.5)	27 (10.9)	70 (10.3)	0.809
d28 mortality	266 (28.9)	60 (24.6)	206 (30.5)	0.084
d90 mortality	323 (37.9)	70 (31.1)	253 (40.3)	0.016

*Data are median (quartile 25%–quartile 75%) or no (%). Percentages may not total 100 because of rounding*.

* *Pulmonary embolism was defined when confirmed by chest CT angiography or when highly suspicious according to clinical assessment and treated accordingly by the attending the physician*.

** *Acute kidney injury was defined when one of the following criteria was met at any point within 28 days after intubation: (1) a 1.5-fold increase of creatinine compared with baseline; and/or (2) an absolute creatinine increase of 26.5 μmol/L compared with baseline; and/or (3) a urinary output <0.5 ml/kg per h for more than 6 h*.

### Ventilation Parameters

**Figure 2:**
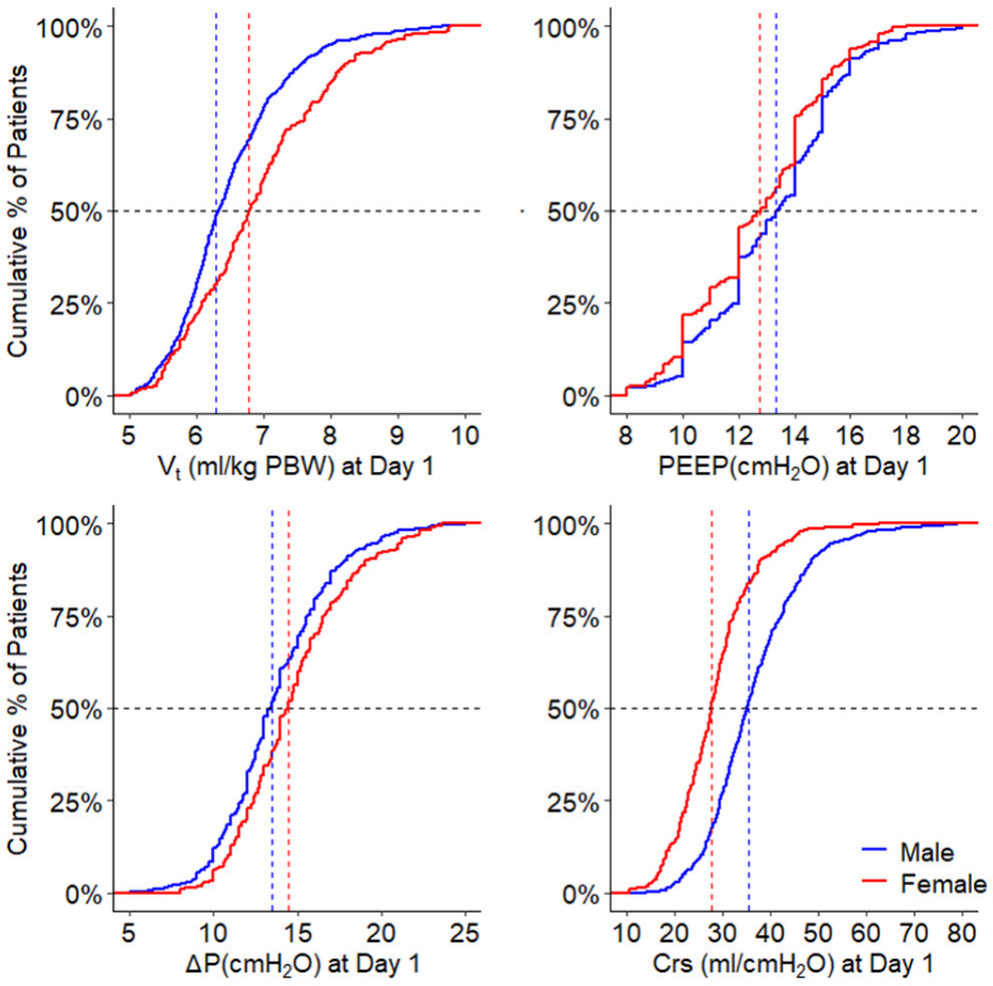
Ventilation parameters during the first day. Cumulative frequency distribution of tidal volume, PEEP, driving pressure, and respiratory system compliance. Vertical dotted lines represent the median on the first calendar day of ventilation for each variable, and horizontal dotted lines show the respective proportion of patients reaching each cutoff. V_T_, tidal volume; PEEP, positive end expiratory pressure; ΔP, driving pressure; Crs, respiratory system compliance; PBW, predicted body weight. The *p*-value for the sex reflects the overall test for difference between sex over the days, the *p*-value for the sex × year interaction evaluates if change over time differed by sex.

**Figure 3:**
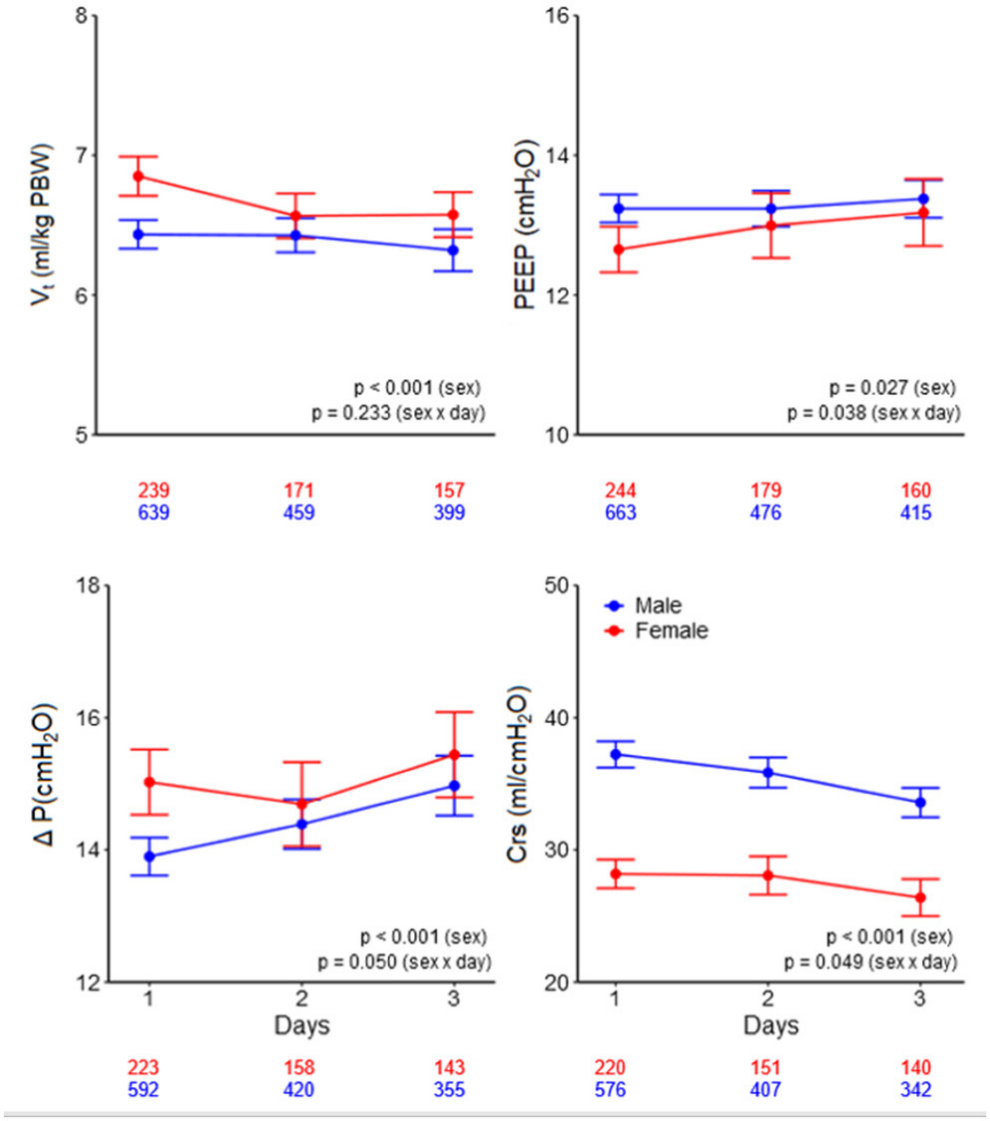
Ventilatory variables over the days. Line graphs with error bars of tidal volume, PEEP, driving pressure, and respiratory system compliance. The numbers under the x-axis indicate the number of patients. V_T_, tidal volume; PEEP, positive end-expiratory pressure; ΔP, driving pressure; Crs, respiratory system compliance; PBW, predicted body weight. The *p*-value for the sex reflects the overall test for difference between sex over the days, the *p*-value for the sex × year interaction evaluates if change over time differed by sex.

On day 1, females received a higher median V_T,PBW_ than males [6.8 (IQR 6.0–7.6, 90% range 5.4–8.8) vs. 6.3 (IQR 5.8–6.9, 90% range 5.0–8.0) ml/kg PBW; *p* < 0.001; Figures 2, 3 and Table 3]. This sex difference became smaller at day 2 [6.4 (IQR 5.9–7.1, 9% range 5.0–8.4) vs. 6.3 (IQR 5.8–7.0, 90% range 5.0–7.9) ml/kg PBW; *p* = 0.046] and at day 3 [6.5 (IQR 6.0–7.1, 90% range 5.1–8.2) vs. 6.2 (IQR 5.6–6.9; 90% range 4.9–7.8) ml/kg PBW; *p* = 0.001; Supplementary Figures 1, 2 and Supplementary Tables 1, 2]. On day 1, females received ventilation with a slightly lower median PEEP and a higher median ΔP (Figures 2, 3 and Table 3). These differences became smaller on days 2 and 3 (Supplementary Figures 1,2 and Supplementary Tables 1, 2). Median Crs was lower in females at all 3 days.

**Table 3:** Ventilatory variables during the first day.

	**Overall**	**Females**	**Males**	***p***
Number of patients	908^*^	244	664	
V_T_, Absolute, mL	451.0 [406.1, 500.0]	396.5 [343.8, 440.5]	468.5 [427.2, 514.8]	<0.001
V_T_, mL/kg ABW	5.2 [4.6, 5.9]	4.9 [4.2, 5.8]	5.3 [4.6, 6.0]	<0.001
90% range	3.4–7.1	3.3–7.0	3.8–7.2	
V_T_, mL/kg PBW	6.4 [5.9, 7.1]	6.8 [6.0, 7.6]	6.3 [5.8, 6.9]	<0.001
90% range	5.1–8.4	5.4–8.8	5.0–8.0	
V_T_, PBW ≤ 6 mL/kg, %	272 (31.0)	56 (23.4)	216 (33.8)	0.003
V_T_, PBW ≤ 8 mL/kg, %	808 (92.0)	202 (84.5)	606 (94.8)	<0.001
V_T_, PBW ≤ 10 mL/kg, %	874 (99.5)	238 (99.6)	636 (99.5)	1.000
PEEP, cmH_2_O	13.2 [11.3, 15.0]	12.7 [10.7, 14.0]	13.3 [11.7, 15.0]	0.002
Peak pressure, cmH_2_O	27.0 [24.0, 30.0]	27.2 [24.7, 30.2]	27.0 [24.0, 30.0]	0.116
Driving pressure, cmH_2_O	13.8 [12.0, 16.0]	14.5 [12.4, 16.9]	13.5 [11.8, 15.8]	<0.001
Mechanical power, J/min	18.9 [15.5, 22.9]	16.8 [14.0, 20.0]	19.8 [16.5, 23.7]	<0.001
Compliance, mL/ cmH_2_O	32.9 [27.5, 40.1]	27.6 [22.6, 32.2]	35.2 [29.6, 42.7]	<0.001
Respiratory rate, bpm	22.0 [20.0, 24.2]	22.0 [20.0, 25.0]	22.0 [20.0, 24.0]	0.257
FiO_2_, %	0.5 [0.4, 0.6]	0.5 [0.4, 0.6]	0.5 [0.4, 0.6]	0.505
SpO_2_, %	95.0 [93.6, 96.4]	95.0 [93.5, 96.2]	95.0 [93.7, 96.5]	0.588
etCO_2_, mmHg	37.5 [33.1, 42.4]	37.0 [32.4, 42.1]	37.5 [33.4, 42.6]	0.351
Heart rate, beats per min	81.0 [70.0, 93.0]	80.4 [71.0, 93.8]	81.1 [69.7, 93.0]	0.713
Mean arterial pressure, mmHg	76.4 [71.5, 82.3]	76.2 [71.9, 82.0]	76.5 [71.3, 82.5]	0.930
pH	7.4 [7.3, 7.4]	7.4 [7.3, 7.4]	7.4 [7.3, 7.4]	0.259
Lactate, mmol/L	1.2 [1.0, 1.5]	1.2 [1.0, 1.5]	1.2 [1.0, 1.5]	0.755
PaO_2_	80.0 [72.9, 90.7]	79.8 [72.2, 91.1]	80.1 [73.0, 89.9]	0.823
P/F ratio	174.2 [142.9, 208.6]	172.7 [135.6, 210.0]	174.6 [145.0, 208.0]	0.549
PaCO_2_, mmHg	45.1 [40.5, 51.2]	44.0 [40.0, 50.1]	45.4 [41.0, 51.8]	0.056
Prone positioning	325 (47.6)	95 (49.2)	230 (46.9)	0.610
Duration of prone positioning	15.0 [11.0, 22.0]	16.0 [11.0, 23.0]	14.0 [11.0, 20.0]	0.129
Minute ventilation	9.7 [8.5, 11.2]	8.6 [7.5, 9.7]	10.2 [8.9, 11.5]	<0.001
Ventilatory ratio	1.7 [1.4, 2.0]	1.7 [1.4, 2.1]	1.7 [1.4, 2.0]	0.030
Recruitment maneuver	16 (2.8)	4 (2.3)	12 (3.0)	0.787

*Data are median (quartile 25%–quartile 75%) or no (%). Percentages may not total 100 because of rounding*.

*PEEP, positive end-expiratory pressure; FiO_2_, fraction of inspired oxygen; SpO_2_, oxygen saturation; PaCO_2_, partial pressure of arterial carbon dioxide; PaO_2_, partial pressure of arterial oxygen*.

* *Of 934 patients who received controlled ventilation on at least one timepoint of data collection, 908 received controlled ventilation at day 1*.

### Use of LTVV

**Figure 4:**
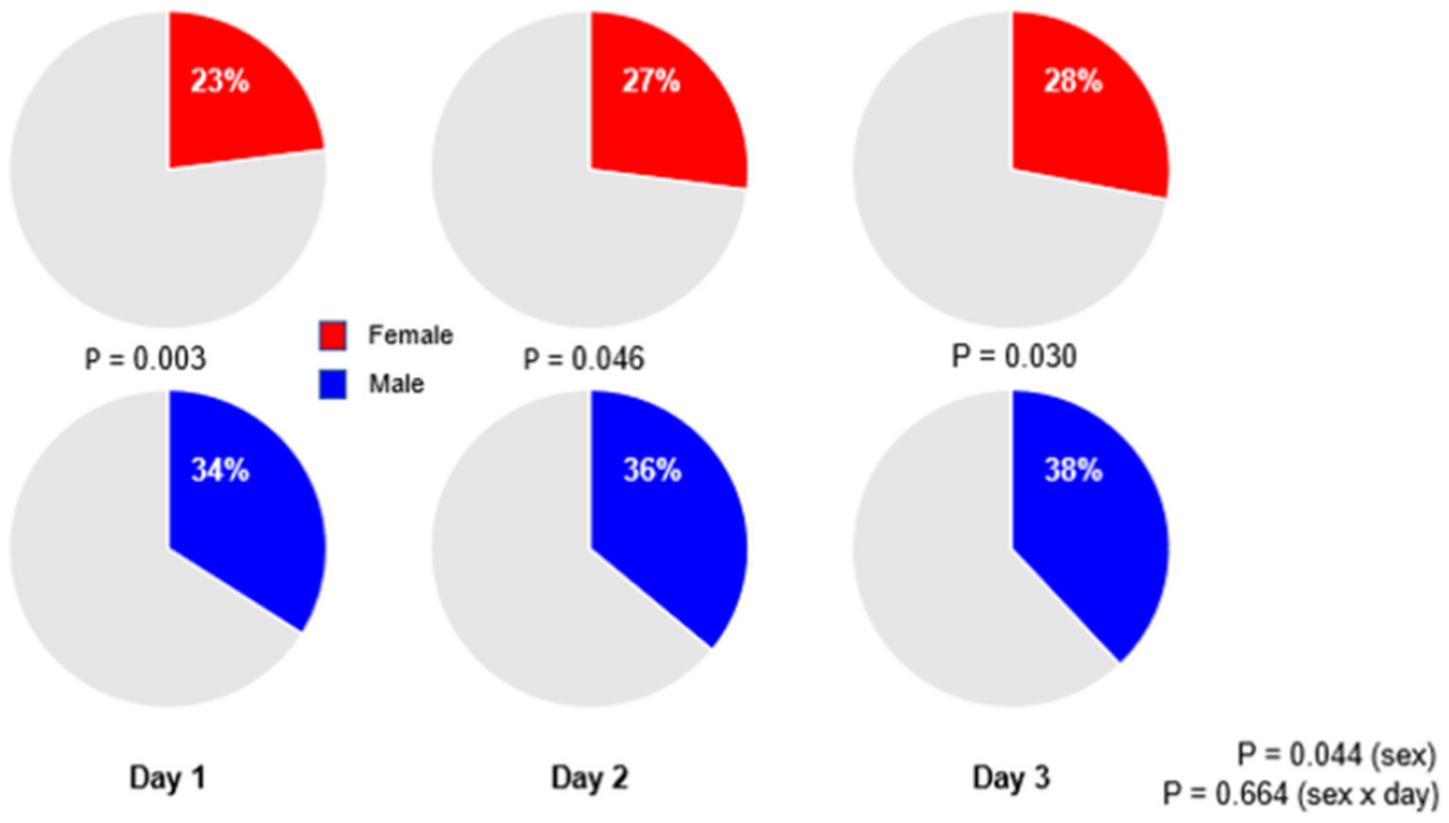
Percentages of patients receiving low tidal volume ventilation. Significant *p*-value for the sex reflects the overall test for difference between sex over the days, while the *p*-value for the sex × year interaction evaluates if change over time differed by sex.

Low tidal volume ventilation was generally underused, with only a third of patients receiving ventilation with a median V_T,PBW_ ≤6 ml/kg PBW–at day 1, females received LTVV less often than males (23 vs. 34%; *p* = 0.003; Figure 4). The sex difference in use of LTVV persisted at day 2 (27 vs. 36%; *p* = 0.046) and at day 3 (28 vs. 38%; *p* = 0.030; Figure 4 and Supplementary Tables 1, 2).

### Mediation Analysis

The difference in the use of LTVV between females and males was significantly mediated by sex [average causal mediation effect 7.5% (95% CI 1.7–13.3%); *p* = 0.011] but more by body height [average causal mediation effect −17.5% (95% CI −21.5 to −13.5%); *p* < 0.001; Table 4]. The difference was also significantly mediated by ABW in the model that only used this factor [average causal mediation effect −1.7% (95% CI −2.7 to −1.0); *p* < 0.001], but not in a model that also used body height, meaning that the difference in the use of LTVV was mainly mediated by differences in height, and not by weight.

**Table 4:** Mediation analysis.

	**Adjusted absolute difference (95% CI)^a^**	***p-value***
**Univariable mediation model**		
**Body height as mediator**		
Total effect of sex	−10.0 (−14.3 to −6.0)	<0.001
Average causal mediation effect of body height	−16.0 (−19.0 to −13.0)	<0.001
Average direct effect of female sex	6.0 (1.0 to 11.0)	0.018
**Body weight as mediator**		
Total effect of sex	−10.0 (−14.3 to −6.0)	<0.001
Average causal mediation effect of body weight	−1.7 (−2.7 to −1.0)	<0.001
Average direct effect of female sex	−8.4 (−12.7 to −4.0)	<0.001
Body height and weight as mediators^*^		
Total effect of sex	−9.8 (−13.6 to −6.0)	<0.001
Average causal mediation effect of body height	−17.5 (−21.5 to −13.5)	<0.001
Average causal mediation effect of body weight	0.2 (−0.8 to 1.2)	0.715
Average direct effect of female sex	7.5 (1.7 to 13.3)	0.011

a *All estimated were generated after 10,000 simulations*.

* *CI estimated from robust clustered standard errors*.

## Discussion

The results of this analysis of a large cohort of critically ill patients with ARDS related to COVID-19 who received invasive ventilation in the ICU during the first wave of the national outbreak in the Netherlands can be summarized as follows: (1) females were at a higher risk of not receiving LTVV at all 4 days of ventilation; (2) PEEP was lower and ΔP was higher, but only at day 1; and (3) females had a lower Crs, a difference that did not change over the days. In addition, the mediation analysis suggests that (4) differences are partly explained by sex *per se*; (5) but are mostly explained by the differences in body height.

Our study has several strengths. The PRoVENT–COVID study is one of the largest multicenter studies that collected ventilator data at several time points per day, allowing a better insight into ventilation practice, and differences herein between females and males. This study involved more than one-third of all invasively ventilated patients with ARDS related to COVID-19 in the first wave of the outbreak in the Netherlands. Furthermore, we enrolled patients in 22 centers included university hospitals, non-university teaching as well as non-teaching hospitals, accounting for around one-fourth of the ICUs in the Netherlands. This all increases the generalizability of the findings. The design of PRoVENT–COVID assured completeness of data collection and the short timeframe within which data were gathered, avoiding the effect of practice changes over time. At last, we followed the analysis plan strictly and used sophisticated mediation analysis to determine which factors determine the sex difference in the use of LTVV.

The differences in V_T_ between females and males may seem small, especially when focusing on the median V_T_, PBW. However, the 90% range clearly shows that V_T_ differs between the sexes–for instance, 16% of female patients received ventilation with a V_T,PBW_ >8 ml/kg, while only 5% of male patients received ventilation with a V_T,PBW_ above this upper threshold of what is generally accepted as safe. The use of a high V_T_ is associated with a higher mortality and morbidity in ICU patients (5, 6, 13–15, 19–21). An earlier analysis showed that a one SD increases in V_T_, _PBW_ meant an increase of 28% in 28-day mortality (5). The finding that females received ventilation with a higher median V_T_ than males in this cohort is in line with results from several investigations originating from before the COVID-19 pandemic (13–16, 44). It interesting to note that V_T_, in both females and males, was lower than in those previous cohorts, suggesting a temporal trend toward the use of lower V_T_ in critically ill patients (16). Despite the improved use of LTVV, however, differences between females and males persist.

Several reports on ventilated in patients with COVID-19 show a higher mortality in male patients (5, 22–30). This was also found in the current cohort. Interestingly, another study reported that the mortality of severely ill premenopausal but not post-menopausal female patients with COVID-19 are lower than age-matched male patients (31). The LUNG SAFE study, before COVID-19, did not find sex differences in mortality, but in that cohort, females had a shorter duration of invasive ventilation and a lower length of ICU stay (15). The reasons why male patients with COVID-19 have higher mortality remains uncertain. Biological factors, hormone factors such as estrogen, and factors related to the activity of X-linked genes have been suggested (31–34) and also sociocultural factors could play a role (34). It could also be interesting to look into the possible benefit of inhalation corticosteroids. In the current cohort, female patients had a significantly higher usage of inhalation corticosteroids as home medication. A total of 2 randomized clinical trials showed that using intravenous corticosteroids could reduce mortality (35, 36). These findings are confirmed in a recent meta-analysis (37). However, it is important to point out the difference between administration, i.e., intravenous vs. inhalation, and setting, i.e., during hospital admission vs. home medication.

The outcome advantage of female patients, however, should not withhold ICU doctors and nurses from using a correct V_T_, seen the advantage of LTVV that has been found in pre-COVID-19 studies and in COVID-19 studies. In fact, this could increase the outcome differences between females and males.

Several studies in patients with non-COVID-19 have shown sex differences in important aspects of care in critically ill patients (7, 38–41). For instance—among patients with sepsis or shock, females are less likely to receive deep venous thrombosis prophylaxis or invasive ventilation, but are more likely to receive red blood cell transfusions (40). On the contrary, males receive “more intense” care, including placement of the central catheters for infusion of vasoactive medication (38) and invasive ventilation (38, 39). It is uncertain if similar differences, i.e., in non-ventilatory care, exist in patients with COVID-19 as well.

Of note, while V_T,PWB_ was higher in the female patients, V_T,ABW_ was higher in the male patients. It should be noticed, however, that the male patients had a significantly higher body mass index (BMI) compared with the females.

Next to the finding that female patients are ventilated with higher V_T,PBW_, it is seen that PEEP was lower and ΔP was higher. These differences were rather small, and probably, therefore, less meaningful, and were only present at day 1. There was a remarkable difference in median Crs between the sexes. This finding is in line with the results of the earlier studies in patients with ARDS before COVID-19 (15, 42). The difference in Crs might be explained by differences in height (42). Further research may reveal associations between other anthropometric factors and Crs.

The findings of the mediation analysis are in line with findings of the previous studies in patients in the ICU (16) and in the operating room (12). In contrary to previous findings, we see that differences are only partly explained by sex *per se*. The actual body weight mediated the sex inequality in the use of LTVV in a model as a single factor, but not in the model using also body height. Our findings point out the importance of using reliable methods to measure the height of the patients.

This analysis has some limitations; first, we only collected data during the first 4 calendar days of ventilation, and we cannot exclude the possibility that ventilation practices beyond these days remain different. Seen the observational nature of the study, we could not control for the unmeasured confounders. Also, the knowledge that ventilation data were being captured could have interfered with daily practice. The selection of ICUs was based on the personal contacts, which could have resulted in an overrepresentation of ICUs with more experience in lung-protective ventilation, including the use of LTVV, and the willingness to participate could have led to selection bias. Another limitation is that because this study was a national study, its worldwide generalizability is uncertain. Finally, the PRoVENT–COVID study did not collect the type of oxygen support before intubation. Early application of HFNC (high-flow nasal cannula) in the mild stage of ARDS may reduce mortality in the elderly patients with severe COVID-19 pneumonia (43). Further research should look into the influence of HFNC and should consider grouping the patients by age to see the impact of age on the use of LTVV in general and in female patients.

## Conclusion

In this cohort of patients with ARDS related to COVID-19 who required invasive ventilation in the first wave of the national outbreak in the Netherlands, females received LTVV less often than males. Alike in the previous studies, in this cohort, the difference in the use of LTVV was driven by the anthropometric factors more than by sex *per se*. This information could be helpful in the proper titration of V_T_ in critically ill patients with COVID-19 and beyond.

## Data Availability Statement

The raw data supporting the conclusions of this article will be made available by the authors, without undue reservation.

## Author Contributions

PS, MS, and FP were involved in the conceptualization, methodology, and worked with drafting of the manuscript. Acquisition, analysis, or interpretation of data was done by all the authors. Critical revision of the manuscript for important intellectual content by all the other authors. Statistical analysis was done by PS and AN. Administrative, technical, or material support was done by MS. MS, FP, and AN supervised the work.

## Funding

This work was supported by Amsterdam University Medical Centers, Location Academic Medical Center, Amsterdam, the Netherlands.

## The Provent–Covid Collaborative Group

Investigators (in alphabetic order) J. P. van Akkeren; A. G. Algera; C. K. Algoe; R. B. van Amstel; O. L. Baur; P. van de Berg; D. C. J. J. Bergmans; D. I. van den Bersselaar; F. A. Bertens; A. J. G. H. Bindels; M. M. de Boer; S. den Boer; L. S. Boers; M. Bogerd; L. D. J. Bos; M. Botta; J. S. Breel; H. de Bruin; S. de Bruin; C. L. Bruna; L. A. Buiteman–Kruizinga; O. Cremer; R. M. Determann; W. Dieperink; D. A. Dongelmans; H. S. Franke; M. S. Galek Aldridge; M. J. de Graaff; L. A. Hagens; J. J. Haringman; N. F. L. Heijnen; S. Hiel; S. T. van der Heide; P. L. J. van der Heiden; L. L. Hoeijmakers; L. Hol; M. W. Hollmann; M. E. Hoogendoorn; J. Horn; R. van der Horst; E. L. K. Ie; D. Ivanov; N. P. Juffermans; E. Kho; E. S. de Klerk; A. W. M. Koopman; M. Koopmans; S. Kucukcelebi; M. A. Kuiper; D. W. de Lange; D. M. van Meenen; Ignacio Martin–Loeches, Guido Mazzinari; N. van Mourik; S. G. Nijbroek; M. Onrust; E. A. N. Oostdijk; F. Paulus; C. J. Pennartz; J. Pillay; L. Pisani; I. M. Purmer; T. C. D. Rettig; J. P Roozeman; M. T. U. Schuijt; M. J. Schultz; A. Serpa Neto; M. E. Sleeswijk; M. R. Smit; P. E. Spronk; W. Stilma; A. C. Strang; A. M. Tsonas; P. R Tuinman; C. M. A. Valk; F. L. Veen; A. P. J. Vlaar; L. I. Veldhuis; P. van Velzen; W. H. van der Ven; P. van Vliet; P. van der Voort; H. H. van der Wier; L. van Welie; H. J. F. T. Wesselink; B. van Wijk; T. Winters; W. Y. Wong; and A. R. H. van Zanten.

## Conflict of Interest

AN reports personal fees from Dräger, outside of the submitted work. MS reports personal fees from the Hamilton Medical, outside of the submitted work. The remaining authors declare that the research was conducted in the absence of any commercial or financial relationships that could be construed as a potential conflict of interest.

## Publisher's Note

All claims expressed in this article are solely those of the authors and do not necessarily represent those of their affiliated organizations, or those of the publisher, the editors and the reviewers. Any product that may be evaluated in this article, or claim that may be made by its manufacturer, is not guaranteed or endorsed by the publisher.
