# Supplementary material for: Sex Differences in Use of Low Tidal Volume Ventilation in COVID-19—Insights From the PRoVENT–COVID Study
Source: Front Med (Lausanne). 2022 Jan 3;8:780005. doi: 10.3389/fmed.2021.780005 (PMC8923734; doi:10.3389/fmed.2021.780005)
Supplement: Supplementary file 1 [file Data_Sheet_1.docx]

Supporting Information with:

**Sex Differences in Use of Protective Ventilation in Invasively Ventilated COVID–19 Patients––insights from the PRoVENT–COVID study**

**Supporting Methods**

**Supporting Tables**

Page 3. eTable 1. Ventilatory variables during the second day

Page 5. eTable 2. Ventilatory variables during the third day

Page 7. eTable 3. Baseline patient characteristics from all patients

Page 10. eTable 4. Outcome from all patients

**Supporting Figures**

Page 12. eFigure 1. Ventilation parameters during the second day

Page 13. eFigure 2. Ventilation parameters during the third day

**eTable 1. Ventilatory variables during the second day**

|  | Overall | Female | Male | p |
| --- | --- | --- | --- | --- |
| Number of patients | 658 | 180 | 478 |  |
| V_T_, Absolute, mL | 446.0 [394.3, 497.0] | 383.7 [347.7, 411.6] | 474.0 [423.7, 510.3] | <0.001 |
| V_T_, mL/kg ABW | 5.1 [4.4, 5.8] | 4.8 [3.9, 5.5] | 5.3 [4.6, 5.9] | <0.001 |
| 90% range | 3.4 – 7.0 | 3.0 – 6.7 | 3.7 – 7.1 |  |
| V_T_, mL/kg PBW | 6.3 [5.8, 7.0] | 6.4 [5.9, 7.1] | 6.3 [5.8, 7.0] | 0.046 |
| 90% range | 5.0 – 8.2 | 5.0 – 8.4 | 5.0 – 7.9 |  |
| V_T_, PBW ≤ 6 mL/kg, % | 209 (33.2) | 46 (26.9) | 163 (35.5) | 0.046 |
| V_T_, PBW ≤ 8 mL/kg, % | 593 (94.1) | 156 (91.2) | 437 (95.2) | 0.084 |
| V_T_, PBW ≤ 10 mL/kg, % | 627 (99.5) | 170 (99.4) | 457 (99.6) | 1.000 |
| PEEP, cmH_2_O | 13.0 [11.3, 15.0] | 12.7 [11.3, 14.7] | 13.7 [11.3, 15.0] | 0.096 |
| Peak Pressure, cmH_2_O | 27.0 [24.0, 30.3] | 27.0 [24.4, 31.0] | 27.0 [24.0, 30.3] | 0.810 |
| Driving Pressure, cmH_2_O | 14.0 [12.0, 16.5] | 14.0 [12.0, 16.6] | 13.7 [11.9, 16.5] | 0.545 |
| Mechanical Power, J/min | 19.8 [15.8, 24.3] | 16.5 [13.8, 20.5] | 21.0 [17.0, 25.4] | <0.001 |
| Compliance, mL/ cmH_2_O | 32.3 [25.9, 39.8] | 27.2 [22.2, 33.1] | 34.0 [28.0, 41.1] | <0.001 |
| Respiratory Rate, bpm | 23.0 [20.0, 26.0] | 23.0 [20.2, 26.0] | 23.0 [20.0, 25.7] | 0.673 |
| FiO_2_, % | 0.4 [0.4, 0.5] | 0.5 [0.4, 0.6] | 0.4 [0.4, 0.5] | 0.405 |
| SpO_2_, % | 94.7 [93.0, 96.0] | 94.3 [93.0, 95.8] | 94.7 [93.3, 96.0] | 0.188 |
| etCO2, mmHg | 38.3 [34.1, 43.9] | 39.0 [35.3, 45.0] | 38.0 [33.8, 43.5] | 0.109 |
| Heartrate, beats per min | 82.0 [70.0, 95.5] | 83.2 [71.2, 96.1] | 81.0 [69.7, 95.0] | 0.261 |
| Mean Arterial Pressure, mmHg | 76.0 [71.3, 81.3] | 75.0 [71.0, 81.7] | 76.5 [71.6, 81.3] | 0.452 |
| pH | 7.4 [7.3, 7.4] | 7.4 [7.3, 7.4] | 7.4 [7.3, 7.4] | 0.432 |
| Lactate, mmol/L | 1.2 [1.0, 1.6] | 1.3 [1.0, 1.7] | 1.2 [1.0, 1.5] | 0.291 |
| PaO2 | 74.3 [68.7, 82.5] | 73.1 [67.5, 84.0] | 74.5 [69.4, 82.2] | 0.505 |
| P/F ratio | 170.6 [141.7, 206.2] | 168.9 [134.1, 207.7] | 171.6 [144.6, 206.2] | 0.406 |
| PaCO2, mmHg | 48.5 [43.5, 54.8] | 48.7 [43.4, 55.3] | 48.5 [43.5, 54.8] | 0.904 |
| Prone positioning | 284 (48.9) | 83 (51.2) | 201 (48.0) | 0.517 |
| Duration of prone positioning | 14.0 [10.0, 21.0] | 12.0 [9.0, 20.0] | 14.0 [11.0, 21.0] | 0.080 |
| Minute Ventilation | 10.0 [8.6, 11.7] | 8.5 [7.7, 9.6] | 10.8 [9.3, 12.1] | <0.001 |
| Ventilatory Ratio | 1.9 [1.6, 2.3] | 1.9 [1.5, 2.3] | 1.8 [1.6, 2.3] | 0.320 |
| Recruitment maneuver | 8 (1.7) | 3 (2.1) | 5 (1.5) | 0.705 |

Data are median (quartile 25% - quartile 75%) or No (%). Percentages may not total 100 because of rounding. PEEP = positive end-expiratory pressure; FiO2 = inspired fraction of oxygen; SpO2 = pulse oximetry; PaCO2 = partial pressure ofcarbon dioxide; PaO2 = partial pressure of oxygen

**eTable 2. Ventilatory variables during the third day**

|  | Overall | Female | Male | p |
| --- | --- | --- | --- | --- |
| Number of patients | 578 | 161 | 417 |  |
| V_T_, Absolute, mL | 436.3 [389.0, 488.0] | 374.7 [343.3, 412.8] | 454.0 [418.5, 505.5] | <0.001 |
| V_T_, mL/kg ABW | 5.0 [4.3, 5.7] | 4.8 [4.0, 5.3] | 5.2 [4.4, 5.8] | <0.001 |
| 90% range | 3.4 – 6.8 | 2.9 – 6.5 | 3.6 – 7.0 |  |
| V_T_, mL/kg PBW | 6.3 [5.7, 6.9] | 6.5 [6.0, 7.1] | 6.2 [5.6, 6.9] | 0.001 |
| 90% range | 4.9 – 8.1 | 5.1 – 8.2 | 4.9 – 7.8 |  |
| V_T_, PBW ≤ 6 mL/kg, % | 196 (35.3) | 44 (28.0) | 152 (38.1) | 0.030 |
| V_T_, PBW ≤ 8 mL/kg, % | 525 (94.4) | 144 (91.7) | 381 (95.5) | 0.100 |
| V_T_, PBW ≤ 10 mL/kg, % | 554 (99.6) | 157 (100.0) | 397 (99.5) | 1.000 |
| PEEP, cmH_2_O | 13.7 [11.6, 15.0] | 13.2 [11.3, 15.0] | 14.0 [12.0, 15.0] | 0.309 |
| Peak Pressure, cmH_2_O | 28.0 [25.0, 31.0] | 28.3 [25.5, 31.0] | 27.7 [25.0, 31.0] | 0.407 |
| Driving Pressure, cmH_2_O | 14.7 [12.3, 17.0] | 15.0 [12.7, 17.3] | 14.5 [12.0, 17.0] | 0.107 |
| Mechanical Power, J/min | 20.1 [16.6, 24.6] | 17.8 [14.7, 20.9] | 21.5 [17.2, 26.2] | <0.001 |
| Compliance, mL/ cmH_2_O | 30.1 [24.3, 36.9] | 25.2 [20.6, 30.7] | 31.8 [26.7, 39.0] | <0.001 |
| Respiratory Rate, bpm | 24.0 [21.0, 26.0] | 24.0 [20.7, 26.0] | 24.0 [21.0, 26.0] | 0.436 |
| FiO_2_, % | 0.5 [0.4, 0.6] | 0.5 [0.4, 0.6] | 0.4 [0.4, 0.6] | 0.077 |
| SpO_2_, % | 94.3 [92.7, 95.5] | 94.3 [92.7, 95.7] | 94.0 [93.0, 95.3] | 0.840 |
| etCO2, mmHg | 39.8 [34.9, 45.5] | 40.1 [35.1, 47.3] | 39.5 [34.8, 45.0] | 0.255 |
| Heartrate, beats per min | 83.7 [72.3, 95.0] | 86.0 [73.0, 97.5] | 83.2 [72.0, 94.0] | 0.096 |
| Mean Arterial Pressure, mmHg | 76.7 [71.3, 82.1] | 76.7 [70.7, 82.3] | 76.7 [72.0, 82.0] | 0.724 |
| pH | 7.4 [7.3, 7.4] | 7.4 [7.3, 7.4] | 7.4 [7.3, 7.4] | 0.332 |
| Lactate, mmol/L | 1.2 [1.0, 1.5] | 1.2 [1.0, 1.5] | 1.2 [1.0, 1.5] | 0.822 |
| PaO2 | 74.3 [67.8, 80.9] | 73.0 [66.8, 81.0] | 74.3 [68.3, 80.6] | 0.349 |
| P/F ratio | 163.1 [131.6, 194.0] | 154.3 [123.4, 191.6] | 165.3 [135.1, 194.7] | 0.067 |
| PaCO2, mmHg | 50.5 [45.7, 57.3] | 51.8 [46.1, 58.5] | 50.3 [45.5, 56.3] | 0.281 |
| Prone positioning | 241 (47.7) | 68 (49.3) | 173 (47.1) | 0.690 |
| Duration of prone positioning | 14.0 [11.0, 19.0] | 16.0 [11.1, 21.5] | 13.0 [11.0, 18.9] | 0.152 |
| Minute Ventilation | 10.2 [8.6, 11.7] | 8.8 [7.7, 10.2] | 10.6 [9.3, 12.3] | <0.001 |
| Ventilatory Ratio | 2.0 [1.7, 2.4] | 2.1 [1.8, 2.6] | 2.0 [1.6, 2.4] | 0.005 |
| Recruitment maneuver | 5 (1.2) | 1 (0.9) | 4 (1.4) | 1.000 |

Data are median (quartile 25% - quartile 75%) or No (%). Percentages may not total 100 because of rounding. PEEP = positive end-expiratory pressure; FiO2 = inspired fraction of oxygen; SpO2 = pulse oximetry; PaCO2 = partial pressure of carbon dioxide; PaO2 = partial pressure of oxygen

**eTable 3. Baseline patient characteristics from all patients**

|  | Overall | Female | Male | p |
| --- | --- | --- | --- | --- |
| Number of patients | 1122 | 305 | 817 |  |
| Age, years | 65.0 [57.0, 72.0] | 65.0 [56.0, 72.0] | 65.0 [58.0, 72.0] | 0.415 |
| Weight, kg | 85.2 [78.0, 96.0] | 79.6 [70.9, 90.0] | 89.0 [80.0, 98.0] | <0.001 |
| Height, cm | 176.0 [170.0, 183.0] | 166.0 [162.0, 172.0] | 180.0 [174.0, 185.0] | <0.001 |
| BMI, kg/m^2^ | 27.7 [25.2, 30.8] | 28.5 [25.8, 32.0] | 27.5 [25.1, 30.1] | 0.001 |
| Intubation at admission | 201 (17.9) | 53 (17.4) | 148 (18.1) | 0.861 |
| NIV before intubation | 85 (8.4) | 25 (8.9) | 60 (8.2) | 0.706 |
| Duration of NIV | 8.0 [2.0, 18.1] | 6.0 [2.0, 13.0] | 8.0 [2.0, 24.0] | 0.481 |
| CT before intubation | 360 (33.6) | 105 (36.3) | 255 (32.6) | 0.274 |
| % affected lung parenchyma on CT |  |  |  | 0.661 |
| 0% | 14 (3.9) | 4 (3.8) | 10 (3.9) |  |
| 25% | 116 (32.0) | 30 (28.6) | 86 (33.3) |  |
| 50% | 107 (29.5) | 37 (35.2) | 70 (27.1) |  |
| 75% | 104 (28.7) | 28 (26.7) | 76 (29.5) |  |
| 100% | 22 (6.1) | 6 (5.7) | 16 (6.2) |  |
| Xray before intubation | 602 (86.1) | 159 (86.4) | 443 (86.0) | 1.000 |
| Number of affected quadrants |  |  |  | 0.371 |
| 1 | 42 (7.0) | 8 (5.1) | 34 (7.6) |  |
| 2 | 139 (23.1) | 43 (27.6) | 96 (21.6) |  |
| 3 | 168 (28.0) | 44 (28.2) | 124 (27.9) |  |
| 4 | 252 (41.9) | 61 (39.1) | 191 (42.9) |  |
| Pneumothorax | 4 (1.4) | 2 (2.8) | 2 (0.9) | 0.262 |
| SAPS II | 36.0 [29.0, 44.0] | 35.0 [30.2, 43.0] | 36.0 [29.0, 44.0] | 0.903 |
| APACHE II | 16.0 [12.0, 20.0] | 15.0 [12.0, 20.0] | 16.0 [13.0, 21.0] | 0.110 |
| APACHE IV | 56.0 [45.0, 69.0] | 55.0 [46.0, 66.0] | 56.9 [44.0, 70.0] | 0.946 |
| SOFA | 7.0 [6.0, 10.0] | 7.0 [6.0, 9.8] | 7.0 [6.0, 10.0] | 0.284 |
| ARDS severity |  |  |  | 0.293 |
| Mild | 224 (20.3) | 56 (18.7) | 168 (20.9) |  |
| Moderate | 759 (68.9) | 204 (68.2) | 555 (69.1) |  |
| Severe | 119 (10.8) | 39 (13.0) | 80 (10.0) |  |
| **Co-existing disorders**    Arterial hypertension | 380 (33.9) | 92 (30.2) | 288 (35.3) | 0.119 |
| Heart failure | 49 (4.4) | 8 (2.6) | 41 (5.0) | 0.100 |
| Diabetes | 250 (22.3) | 62 (20.3) | 188 (23.0) | 0.375 |
| Chronic kidney disease | 47 (4.2) | 10 (3.3) | 37 (4.5) | 0.406 |
| Baseline creatinine, μmol/L* | 77.0 [63.0, 97.0] | 63.0 [52.0, 77.8] | 82.0 [69.0, 102.0] | <0.001 |
| Liver cirrhosis | 3 (0.3) | 2 (0.7) | 1 (0.1) | 0.181 |
| COPD | 88 (7.8) | 28 (9.2) | 60 (7.3) | 0.319 |
| Active hematological malignancy | 16 (1.4) | 3 (1.0) | 13 (1.6) | 0.579 |
| Active solid tumor malignancy | 28 (2.5) | 10 (3.3) | 18 (2.2) | 0.290 |
| Neuromuscular disease | 8 (0.7) | 3 (1.0) | 5 (0.6) | 0.455 |
| Immunosuppression | 24 (2.1) | 7 (2.3) | 17 (2.1) | 0.818 |
| **Home medication**  Systemic corticosteroids | 38 (3.4) | 9 (3.0) | 29 (3.5) | 0.713 |
| Inhalation corticosteroids | 125 (11.1) | 47 (15.4) | 78 (9.5) | 0.007 |
| ACE inhibitor | 189 (16.8) | 46 (15.1) | 143 (17.5) | 0.370 |
| ARB II | 127 (11.3) | 26 (8.5) | 101 (12.4) | 0.073 |
| Beta blocker | 211 (18.8) | 48 (15.7) | 163 (20.0) | 0.122 |
| Insulin | 78 (7.0) | 23 (7.5) | 55 (6.7) | 0.692 |
| Metformin | 175 (15.6) | 38 (12.5) | 137 (16.8) | 0.079 |
| Statin | 330 (29.4) | 69 (22.6) | 261 (31.9) | 0.002 |
| Calcium channel blocker | 197 (17.6) | 47 (15.4) | 150 (18.4) | 0.290 |

Data are median (quartile 25% - quartile 75%) or No (%). Percentages may not total 100 because of rounding

*Most recent measurement in 24 hours before intubation, or at ICU admission under invasive ventilation

APACHE = Acute Physiology and Chronic Health Evaluation; SAPS =: Simplified Acute Physiology Score; SOFA = Sequential

Organ Failure Assessment; ARDS = acute respiratory distress syndrome; CT = computed tomography

**eTable 4. Outcome from all patients**

|  | Overall | Female | Male | p |
| --- | --- | --- | --- | --- |
| Number of patients | 1122 | 305 | 817 |  |
| Ventilatory free days at 28 days | 2.0 [0.0, 16.0] | 9.0 [0.0, 18.0] | 0.0 [0.0, 15.0] | <0.001 |
| Extubation | 662 (59.4) | 191 (62.6) | 471 (58.1) | 0.194 |
| Duration of ventilation, days | 14.0 [8.0, 23.0] | 12.0 [7.0, 19.0] | 15.0 [8.0, 25.0] | 0.001 |
| Duration of ventilation in survivors at day 28 | 16.0 [10.0, 28.0] | 13.0 [9.0, 23.0] | 17.0 [10.0, 30.5] | <0.001 |
| Tracheostomy | 190 (17.1) | 37 (12.2) | 153 (18.9) | 0.007 |
| Reintubation | 140 (12.6) | 42 (13.9) | 98 (12.2) | 0.478 |
| Pneumothorax | 9 (0.8) | 1 (0.3) | 8 (1.0) | 0.458 |
| Thromboembolic complications* | 319 (28.4) | 81 (26.6) | 238 (29.1) | 0.414 |
| Acute kidney injury** | 496 (44.4) | 116 (38.3) | 380 (46.6) | 0.015 |
| Renal replacement therapy | 205 (18.3) | 39 (12.8) | 166 (20.3) | 0.003 |
| ICU length of stay, days | 15.0 [9.0, 26.0] | 14.0 [8.5, 23.0] | 16.0 [9.0, 27.0] | 0.015 |
| In survivors, days | 17.0 [10.0, 29.0] | 15.0 [10.0, 26.0] | 18.0 [11.0, 31.0] | 0.021 |
| Hospital length of stay, days | 23.0 [14.0, 37.0] | 22.0 [14.0, 33.3] | 24.0 [14.0, 38.0] | 0.171 |
| In survivors, days | 29.0 [20.0, 44.0] | 27.0 [20.0, 39.0] | 30.0 [20.0, 47.0] | 0.029 |
| ICU mortality | 356 (32.6) | 82 (27.9) | 274 (34.4) | 0.049 |
| Hospital mortality | 367 (35.9) | 83 (30.6) | 284 (37.8) | 0.039 |
| d7 mortality | 116 (10.4) | 35 (11.6) | 81 (10.0) | 0.440 |
| d28 mortality | 318 (28.9) | 77 (25.9) | 241 (29.9) | 0.203 |
| d90 mortality | 383 (37.7) | 87 (31.9) | 296 (39.9) | 0.020 |

Data are median (quartile 25% - quartile 75%) or No (%). Percentages may not total 100 because of rounding.

* Pulmonary embolism was defined when confirmed by chest CT angiography or when

highly suspicious according to clinical assessment and treated accordingly by the

attending physician

** Acute kidney injury was defined when one of the following criteria was met at any

point within 28 days after intubation: 1) a 1.5-fold increase of creatinine compared to

baseline; and/or 2) an absolute creatinine increase of 26.5 μmol/L compared to

baseline; and/or) a urinary output <0.5 mL/kg per hour for more than 6 hours.

**eFigure 1. Ventilation parameters during the second day**

**
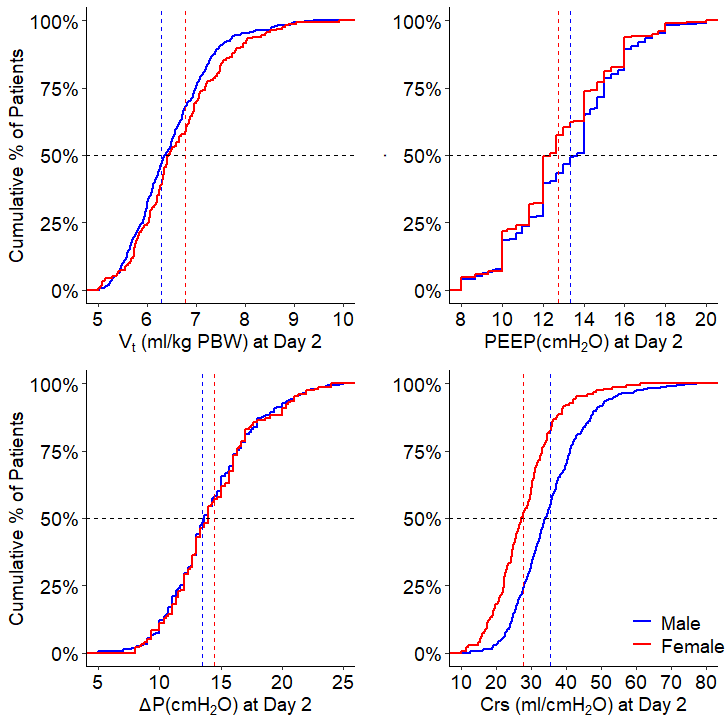
**

Cumulative frequency distribution of tidal volume, PEEP, driving pressure, and respiratory system compliance. Vertical dotted lines represent the median on the first calendar day of ventilation for each variable, and horizontal dotted lines show the respective proportion of patients reaching each cutoff.

V_T_, tidal volume; PEEP, positive end–expiratory pressure; ΔP, driving pressure; Crs, respiratory system compliance; PBW, predicted bodyweight. The P value for the sex reflects the overall test for difference between sex over the days, the P value for the sex x year interaction evaluates if change over time differed by sex.

**eFigure 2. Ventilatory variables during the third day**

**
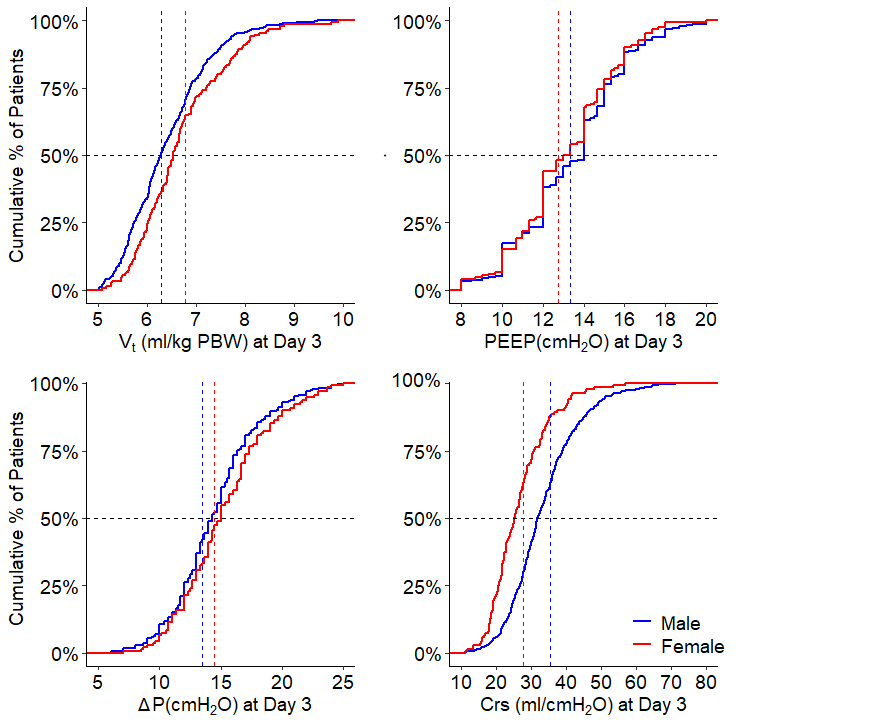
**

Cumulative frequency distribution of tidal volume, PEEP, driving pressure, and respiratory system compliance. Vertical dotted lines represent the median on the first calendar day of ventilation for each variable, and horizontal dotted lines show the respective proportion of patients reaching each cutoff.

V_T_, tidal volume; PEEP, positive end–expiratory pressure; ΔP, driving pressure; Crs, respiratory system compliance; PBW, predicted bodyweight. The P value for the sex reflects the overall test for difference between sex over the days, the P value for the sex x year interaction evaluates if change over time differed by sex.
